# Propionate Protects against Lipopolysaccharide-Induced Mastitis in Mice by Restoring Blood–Milk Barrier Disruption and Suppressing Inflammatory Response

**DOI:** 10.3389/fimmu.2017.01108

**Published:** 2017-09-15

**Authors:** Jingjing Wang, Zhengkai Wei, Xu Zhang, Yanan Wang, Zhengtao Yang, Yunhe Fu

**Affiliations:** ^1^College of Veterinary Medicine, Jilin University, Changchun, China

**Keywords:** mastitis, sodium propionate, blood–milk barrier, NF-κB, histone deacetylases

## Abstract

Mastitis, an inflammation of the mammary glands, is a major disease affecting dairy animal worldwide. Propionate is one of the main short-chain fatty acid that can exert multiple effects on the inflammatory process. The purpose of this study is to investigate the mechanisms underlying the protective effects of sodium propionate against lipopolysaccharide (LPS)-induced mastitis model in mice. The data mainly confirm that inflammation and blood–milk barrier breakdown contribute to progression of the disease in this model. In mice with LPS, sodium propionate attenuates the LPS-induced histopathological changes, inflammatory cytokines tumor necrosis factor-α (TNF-α), interleukin-6 (IL-6), and interleukin-1β (IL-1β) production, myeloperoxidase activity in mammary tissues. Given their importance in the blood–milk barrier, tight junction proteins occludin and claudin-3 are further investigated. Our results show that sodium propionate strikingly increases the expressions of occludin and claudin-3 and reduces the blood–milk barrier permeability in this model. Furthermore, in LPS-stimulated mouse mammary epithelial cells (mMECs), LPS increased the expressions of phosphorylated (p)-p65, p-IκB proteins, which is attenuated by sodium propionate. Finally, we examine the possibility that propionate acts as a histone deacetylase (HDAC) inhibitor, the results show that both sodium propionate and trichostatin A increase the level of histone H3 acetylation and inhibit the increased production of TNF-α, IL-6, and IL-1β in LPS-stimulated mMECs. These data suggest that sodium propionate protects against LPS-induced mastitis mainly by restoring blood–milk barrier disruption and suppressing inflammation *via* NF-κB signaling pathway and HDAC inhibition.

## Introduction

Mastitis, an inflammation of the mammary glands, is usually caused by bacterial pathogens invading the mammary glands. Lipopolysaccharide (LPS) is a powerful bacterial virulence factor typically associated with acute clinical mastitis (1). Furthermore, during mammary gland inflammation, the blood–milk barrier can become leaky and the molecules can across the barrier into milk and *vice versa* (2, 3). Bacterial infection also causes the disruption of directionally controlled milk secretion. For example, concentration of serum albumin increases in milk during mastitis because of an alteration in the blood–milk barrier. Therefore, serum albumin concentrations in milk can be used as an indicator of permeability of the blood–milk barrier (4). Therefore, maintenance of integrity of the blood–milk barrier may hold potential therapeutic benefit for the treatment of inflammation.

Integrity of the blood–milk barrier of alveolar epithelium is maintained by alveolar epithelial tight junctions (TJs) that block the leakage of milk components from the luminal side into the blood serum. In the mammary gland, the less permeable TJs are established shortly after parturition and that remains closed throughout lactation (5, 6). Intramammary administration of LPS changes the composition of the TJ proteins, which is associated with the disruption of the blood–milk barrier (7). These proteins form a junction between the actin cytoskeleton and transmembrane proteins and are attributed to form a tight connection between epithelial cells that represents the blood–milk barrier. Short-chain fatty acids (SCFAs) can alter TJ permeability in human umbilical vein endothelial cells (8). In this study, we investigate the effects of sodium propionate on integrity of the blood–milk barrier.

Dietary fibers are complex carbohydrates, which serve as substrates for anaerobic fermentation that produce three major luminal SCFAs, including acetate, propionate, and butyrate, as end products (9). SCFAs readily reach millimolar concentrations in the colonic lumen (10), with butyrate, propionate, and acetate in a molar ratio approximately 15:25:60, respectively. The beneficial effects of SCFAs on various aspects of gut physiology, barrier function, and metabolism have been well documented (11). Furthermore, SCFAs can promote intestinal homeostasis and suppress intestinal inflammation (12, 13). Recently, several reports have been published describing inhibitory effects of SCFA on NF-κB, one of the key transcription factors regulating genes implicated in innate immunity, cell cycle control, and apoptosis (14). However, most previous studies mainly focused on butyrate, and few studies have devoted their efforts to other SCFAs such as propionate, although it is abundant as butyrate in the gut and blood. Thus, the purpose of this study is to investigate protective mechanisms of sodium propionate in LPS-induced mastitis model.

Biochemically, it has been reported butyrate and propionate act as histone deacetylase (HDAC) inhibitors (15, 16). Recently, the anti-inflammatory effects of HDAC inhibitors have attracted much attention. HDAC inhibitors have been reported to regulate the activity of the transcription factor NF-κB in number of different cell types (17, 18). NF-κB is an essential transcription factor that is strongly associated with regulate inflammatory and immune responses to extracellular stimulus (19–21). Upon activation, NF-κB rapidly enhances the expression of pro-inflammatory genes. The ability of propionate and other HDAC inhibitors to modulate NF-κB activity coincides with its proposed cancer suppressing and anti-inflammatory activities. In this study, we mainly focus on sodium propionate regulation of inflammatory responses and underlying mechanisms.

## Materials and Methods

### Animals

Pregnant BALB/c mice were purchased from the Center of Experimental Animals of Baiqiuen Medical College of Jilin University (Jilin, China). All animal experiments were performed to the Guide for the Care and Use of Laboratory Animals from the National Institutes of Health and were approved by the Animal Care and Use Committee of Wenzhou University. All animals were housed in standard temperature conditions with 12:12 h light–dark cycle and fed with food and water.

On day 10 of lactation, the lactating mice that were kept with suckling neonatal pups are randomly selected. The mice were divided into the following five groups: the blank control group, LPS treatment group, LPS + sodium propionate (50, 100, and 200 mg/kg) treatment groups. Sodium propionate (50, 100, and 200 mg/kg) was intraperitoneally given before LPS administration. For the blank control group, mice were given an equal volume of sterile saline. After 1 h, LPS (0.2 mg/ml) was injected into the fourth inguinal mammary gland. 24 h after LPS injection, the mice were sacrificed, and the mammary glands were collected.

### Histological Analysis

Mammary tissues were fixed in 4% paraformaldehyde solution immediately and embedded in paraffin and cut into 5-μm thick sections. For the evaluation of histopathology changes, sections were stained with hematoxylin–eosin (H&E) and observed under a microscope.

### Immunofluorescence Analysis

The paraformaldehyde-fixed mammary glands were embedded in paraffin and cut into 5-μm thick slices. The slices were incubated with fluorescein isothiocyanate (FITC) albumin (2.5 mg/ml) overnight at 4°C after blocking with 5% goat serum for 40 min. After the slices were washed with PBS, they were treated with an appropriate secondary antibody for 1 h at room temperature in the blocking solution. After washing with PBS three times, the slices were stained with 4′,6-diamidino-2-phenylindole dihydrochloride (DAPI) and mounted using the fluorescence microscope.

### Enzyme-Linked Immunosorbent Assay (ELISA) and Myeloperoxidase (MPO) Activity Assay

Concentrations of tumor necrosis factor-α (TNF-α), interleukin-6 (IL-6), and interleukin-1β (IL-1β) levels in mammary glands supernatant were determined using the mouse ELISA kits (eBioscience, San Diego, CA, USA) according to the manufacturer’s instructions. MPO activity was assessed by ELISA kits (Nanjing Jiancheng Bioengineering Institute, China) according to the manufacturer’s instructions.

### Cell Culture

Mouse mammary epithelial cells (mMECs) were purchased from ATCC (ATCC^®^ CRL-3063™) and cultured in DMEM F12 medium (Hyclone) supplemented with 10% (v/v) fetal bovine serum (CLARK) and antibiotics (100 U/ml penicillin and 100 µg/ml streptomycin sulfate) (Hyclone) at 37°C humidified incubator 5% CO_2_.

### Cell Viability Assay

The effect of sodium propionate on cell viability was determined using the MTT assay. MMECs (4 × 10^5^ cells/ml) were plated in 96-well plates at 37°C for 1 h. The cells were subsequently treated with sodium propionate (0.1–2 mM) for 18 h in the presence or absence of LPS (1 µg/ml). Then, 20 µl of MTT (5 mg/ml) was added for 4 h. The supernatant was removed, and 150 µl/well of dimethylsulfoxide was added. The attenuance was read at 570 nm using a microplate reader.

### Real-time PCR

Cells were pretreated with sodium propionate (0.1, 0.25, 0.5, and 1 mM) or trichostatin A (TSA) (6.25, 12.5, 25, and 50 nM) for 12 h followed by incubation with 1 µg/ml LPS for 3 h. Total RNA was isolated from the mMECs using Trizol Reagent (Invitrogen, Carlsbad, CA, USA) and reverse transcribed using RevertAid First Strand cDNA Synthesis Kit (Thermo Fisher Scientific) according to the manufacturer’s protocol. The polymerase chain reaction was performed at 50°C for 2 min, at 95°C for 10 min, at 95°C for 15 s, and at 60°C for 1 min, followed by 40 cycles. The following primer sequences are designed in Table 1, and the gene expression levels were analyzed with the 2^−ΔΔCT^.

**Table 1:** Primers used in this study.

Gene	Primer	Sequence 5′ > 3′	Product size (bp)
Tumor necrosis factor-α-α	Sense	GCCTCCCTCTCATCAGTTCTA	246
	Anti-sense	GGCAGCCTTGTCCCTTG	
Interleukin-1β	Sense	ACCTGTGTCTTTCCCGTGG	162
	Anti-sense	TCATCTCGGAGCCTGTAGTG	
Interleukin-6	Sense	AGTTGTGCAATGGCAATTCTGA	223
	Anti-sense	CCCCAGCATCGAAGGTAGA	
GAPDH	Sense	TGCTGTCCCTGTATGCCTCT	
	Anti-sense	TTTGATGTCACGCACGATTT	224
β-actin	Sense	TCACCAACTGGGACGACA	
	Anti-sense	GCATACAGGGACAGCACA	206

### Western Blot Analysis

Samples of the mammary tissues and mMECs were obtained, and the whole proteins were extracted with a total protein extraction kit (Thermo Fisher Scientific) according to the manufacturer’s instructions. The protein concentrations were determined using the BCA assay kit. Next, each specimen with amount 50 µg total protein was separated by 10% SDS–polyacrylamide gel electrophoresis and transferred onto polyvinylidene difluoride membranes. The membranes were blocked for 2 h with TBS containing 5% non-fat dried milk and 0.05% Tween-20 at room temperature. Subsequently, the members were incubated overnight at 4°C with primary antibodies. Finally, the membranes were washed in TBS containing 0.05% Tween-20 and incubated for 2 h at room temperature with appropriate secondary HRP-conjugated antibodies. Specific bands were visualized with an ECL detection kit (Thermo Fisher Scientific).

### Statistical Analysis

All data were evaluated using the Statistical Analysis System (GraphPad InStat Software, San Diego, CA, USA). The data were expressed as mean ± SEM from three or more independent experiments. Statistical analysis was performed using one-way analysis of variance and Dunnett’s test. Statistical significance was set to *p* < 0.05 or *p* < 0.01.

## Results

### Effects of Sodium Propionate of Histopathology in LPS-Induced Mastitis in Mice

**Figure 1:**
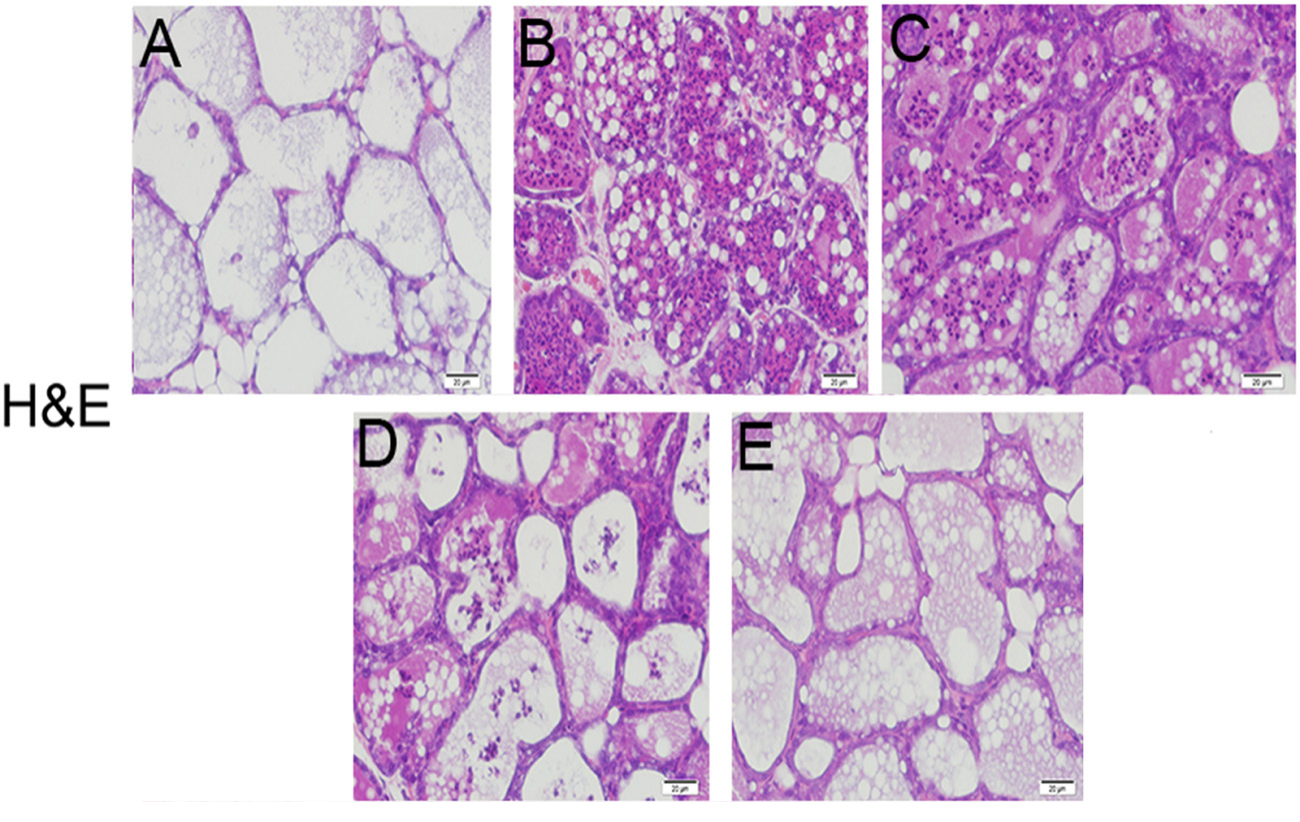
Effects of sodium propionate on lipopolysaccharide (LPS)-induced histopathological impairment of mammary glands. Representative images of LPS-induced mammary glands **(A)** the control group, **(B)** LPS-treated group, **(C)** LPS + sodium propionate (50 mg/kg) group, **(D)** LPS + sodium propionate (100 mg/kg) group, and **(E)** LPS + sodium propionate (200 mg/kg) group. Original magnification, 200×.

The histological and morphological characteristics of the mammary glands were assessed by H&E staining (Figure 1). There were no inflammatory reactions on the mammary glands from the blank control group (Figure 1A). However, the mammary glands from LPS group (Figure 1B) presented serious histopathological changes, represented by thickening of the alveolar wall, hyperemia, interstitial patchy hemorrhage, edema, and the extensive existence of inflammatory cells in alveolar spaces. Compared with LPS group, sodium propionate (Figures 1C–E) ameliorated LPS-induced histopathological changes in a dose-dependent manner. These results suggest that sodium propionate could ameliorate mammary tissues injury induced by LPS.

### Effects of Sodium Propionate on the MPO Activity

**Figure 2:**
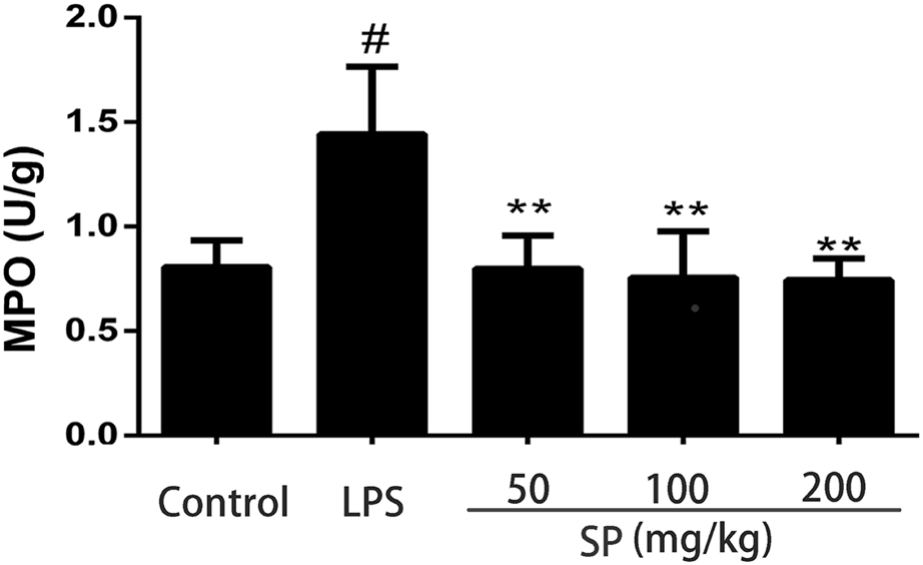
Effects of sodium propionate on myeloperoxidase (MPO) activity. Results are given as mean ± SEM. Differences between groups were determined by one-way analysis of variance followed by Duncan’s *post hoc* analysis. ^#^*p* < 0.05 versus the control group, and *p < 0.05, **p < 0.01 versus the lipopolysaccharide (LPS)-treated group.

The accumulation of activated neutrophils was assessed by determining MPO activity in the mammary glands. As shown in Figure 2, the LPS group showed a significant increase of MPO activity compared with the blank control group. MPO activity was markedly reduced in the treatment groups in comparison with the LPS group.

### Effects of Sodium Propionate on Cytokines Production in LPS-Induced Mastitis Model

**Figure 3:**
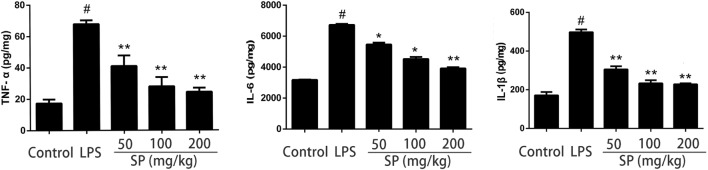
Effects of sodium propionate on cytokines levels in mammary glands. Levels of tumor necrosis factor-α (TNF-α), interleukin-1β (IL-1β), and interleukin-6 (IL-6) were measured by enzyme-linked immunosorbent assay. Results are given as mean ± SEM. Differences between groups were determined by one-way analysis of variance followed by Duncan’s *post hoc* analysis. ^#^*p* < 0.05 versus the control group, and *p < 0.05, **p < 0.01 versus the lipopolysaccharide (LPS)-treated group.

Cytokines TNF-α, IL-6, and IL-1β levels were determined using ELISA (Figure 3). LPS stimulation leading to a significant production of all cytokines tested, and sodium propionate suppressed the production of these cytokines in mammary glands.

### Effects of Sodium Propionate on Blood–Milk Barrier Functions in LPS-Induced Mastitis Model

**Figure 4:**
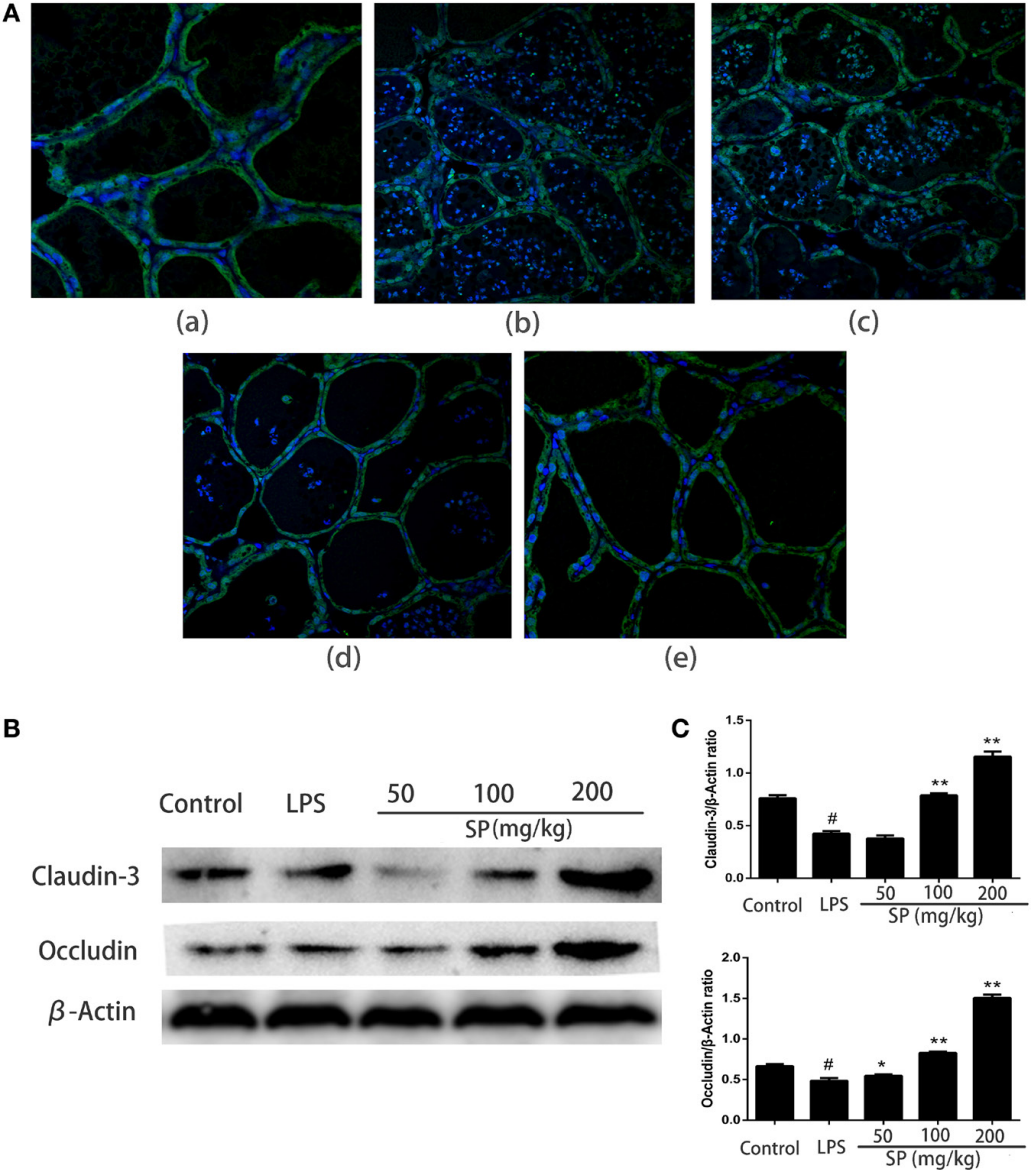
Protective effects of sodium propionate on the function of the blood–milk barrier in lipopolysaccharide (LPS)-induced mastitis. **(A)** Representative images of the fluorescein isothiocyanate (FITC) albumin staining in different group. Green and blue show FITC albumin and nuclei (4′,6-diamidino-2-phenylindole dihydrochloride, DAPI), respectively. [**(A)**, a] the control group, [**(A)**, b] LPS-treated group, [**(A)**, c] LPS + sodium propionate (50 mg/kg) group, [**(A)**, d] LPS + sodium propionate (100 mg/kg) group, and [**(A)**, e] LPS + sodium propionate (200 mg/kg) group. Original magnification, 400×. **(B)** Representative western blots showed expression of claudin-3 and occludin. **(C)** Quantification of claudin-3 and occludin was determined by densitometry and is normalized to β-actin. Results are given as mean ± SEM. Differences between groups were determined by one-way analysis of variance followed by Duncan’s *post hoc* analysis. ^#^*p* < 0.05 versus the control group, and *p < 0.05, **p < 0.01 versus the LPS-treated group.

To evaluated the influence of sodium propionate on the blood–milk barrier. The permeability of the blood–milk barrier was analyzed by FITC albumin leakage assay (Figure 4A). FITC albumin was localized on the interstitial side after saline injection. 24 h after injection of LPS induced some leakage of FITC albumin from the interstitial side into the alveolar lumen. However, pretreatment with sodium propionate can restore the fluorescent reactions.

To understand how sodium propionate reduces the disruption of the blood–milk barrier, we detected the effects of sodium propionate on the TJ proteins expressions (Figures 4B,C). Compared with LPS group, sodium propionate significantly increases the protein levels of occludin and claudin-3, indicating that sodium propionate might contribute to the protection of the blood–milk barrier function.

### Effects of Sodium Propionate on Cell Viability

**Figure 5:**
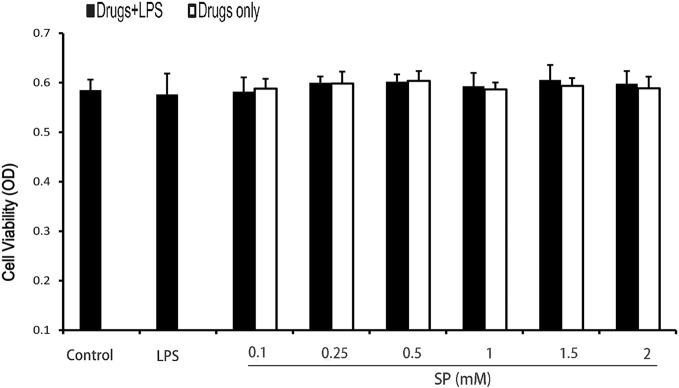
Effects of sodium propionate on cell viability. Cells were cultured with different concentrations of sodium propionate (0.125, 0.25, 0.5, 1, 1.5, and 2 mM) in the presence or absence of 1 µg/ml lipopolysaccharide (LPS) for 18 h. The cell viability was determined by MTT assay. Results are given as mean ± SEM. Differences between groups were determined by one-way analysis of variance followed by Duncan’s *post hoc* analysis. ^#^*p* < 0.05 versus the control group, and *p < 0.05, **p < 0.01 versus the LPS-treated group.

The potential cytotoxicity of sodium propionate was evaluated by the MTT assay after incubating cells for 18 h in the presence or absence of LPS (1 µg/ml). The results show that cell viabilities are not affected by the sodium propionate at the concentrations used (0.1–2 mM) (Figure 5). Thus, the effects of sodium propionate on mMECs were not attributable to cytotoxic effects.

### Effects of Sodium Propionate and HDAC Inhibitors on Cytokines production in LPS-Stimulated mMECs

**Figure 6:**
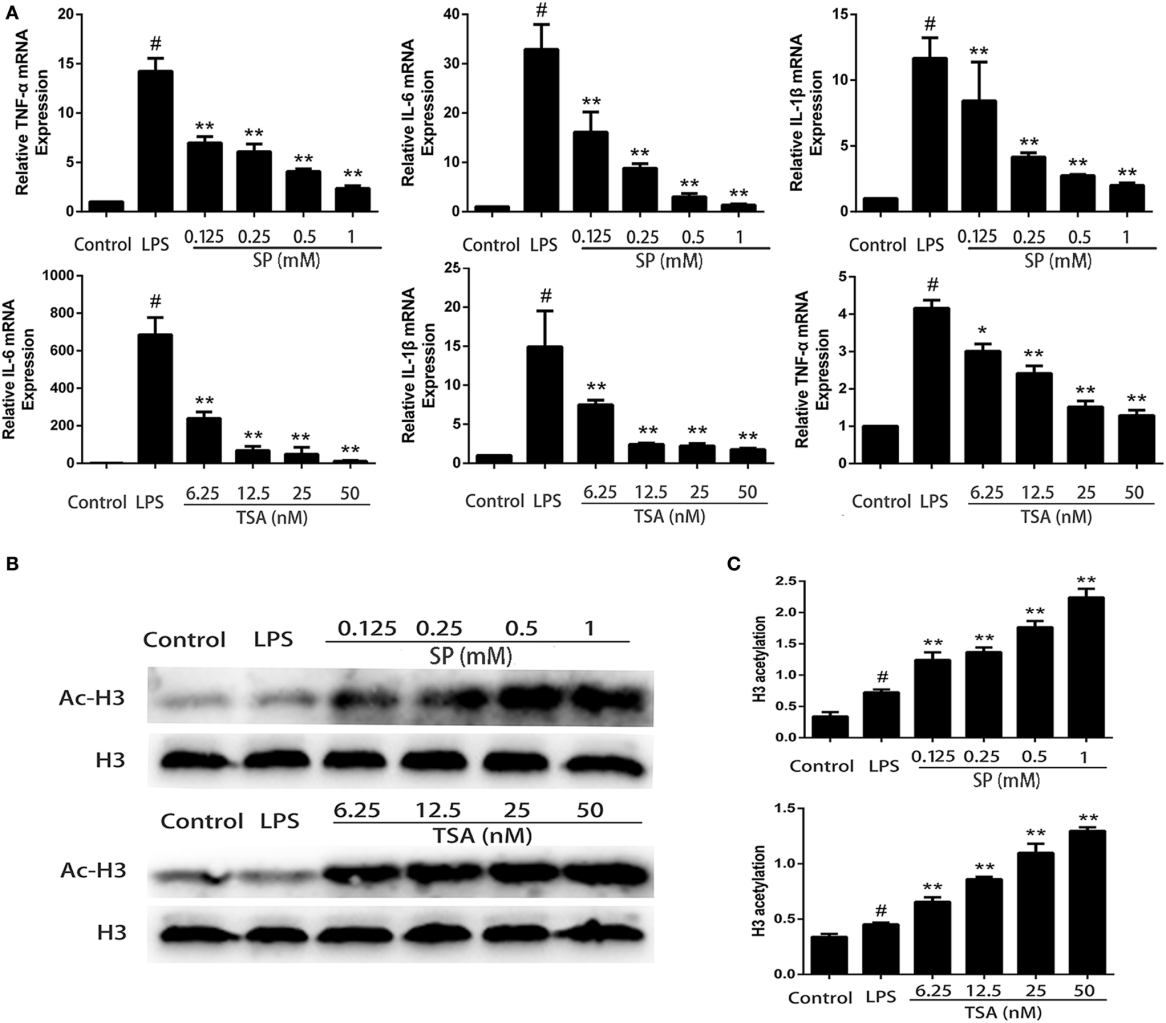
Sodium propionate acts as a histone deacetylase inhibitor in mouse mammary epithelial cells (mMECs). **(A)** Effects of sodium propionate and trichostatin A (TSA) on cell cytokine production in lipopolysaccharide (LPS)-induced mMECs. Cells were pretreated with sodium propionate (0.1, 0.25, 0.5, and 1 mM) or TSA (6.25, 12.5, 25, and 50 nM) for 12 h followed by incubation with 1 µg/ml LPS for 3 h. Levels of tumor necrosis factor-α (TNF-α), interleukin-1β (IL-1β), and interleukin-6 (IL-6) were measured by qRT-PCR. **(B)** Western blot analysis of the acetylated histone H3 was determined. **(C)** Quantification of H3 acetylation was determined by densitometry and is normalized to β-actin. Results are given as mean ± SEM. Differences between groups were determined by one-way analysis of variance followed by Duncan’s *post hoc* analysis. ^#^*p* < 0.05 versus the control group, and *p < 0.05, **p < 0.01 versus the LPS-treated group.

We investigate whether propionate exerts anti-inflammatory effects on LPS-stimulated mMECs. Cells were pretreatment with sodium propionate and TSA (a HDAC inhibitor) for 12 h followed by incubation with 1 µg/ml LPS for 3 h. The expressions of TNF-α, IL-6, and IL-1β are determined by qRT-PCR. The results show that both sodium propionate and TSA suppress TNF-α, IL-6, and IL-1β production in LPS-stimulated mMECs in a dose-dependent manner (Figure 6A). To further demonstrate that sodium propionate acts as a HDAC inhibitor, we quantify the histone H3 acetylation levels by western blotting. Similar to TSA, sodium propionate increase histone acetylation (Figure 6B). These results suggest that sodium propionate behaves as a HDAC inhibitor in mMECs.

### Effects of Sodium Propionate on NF-κB Signaling Pathway in LPS-Stimulated mMECs

**Figure 7:**
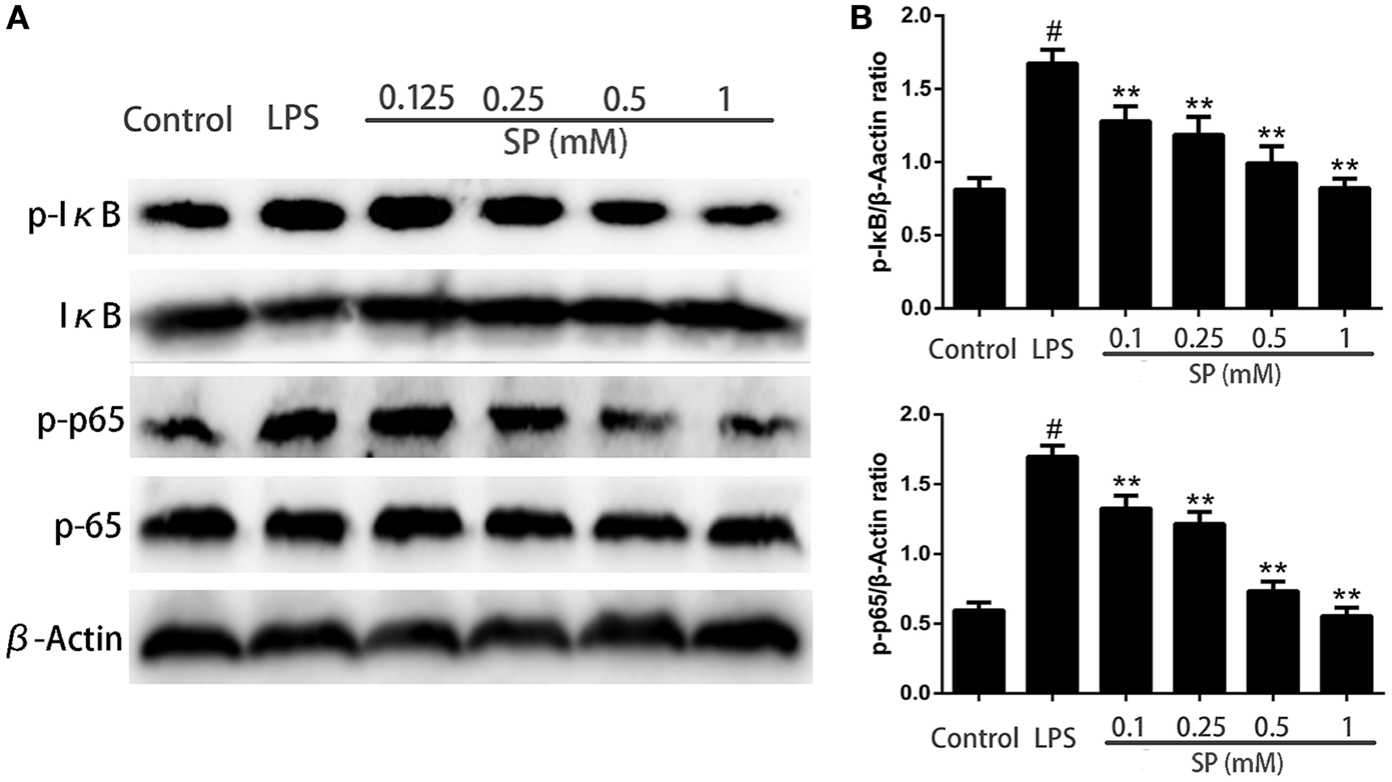
Effects of sodium propionate on NF-κB signaling pathway in lipopolysaccharide (LPS)-induced mouse mammary epithelial cells. Cells were pretreated with sodium propionate (0.1, 0.25, 0.5, and 1 mM) for 12 h followed by incubation with 1 µg/ml LPS for 3 h. **(A)** Protein samples were analyzed by western blotting with specific antibodies. **(B)** Quantification of protein samples was determined by densitometry and is normalized to β-actin. Results are given as mean ± SEM. Differences between groups were determined by one-way analysis of variance followed by Duncan’s *post hoc* analysis. ^#^*p* < 0.05 versus the control group, and *p < 0.05, **p < 0.01 versus the LPS-treated group.

To investigate whether NF-κB is involved in the pathway through which sodium propionate regulates inflammation and the blood–milk barrier function. NF-κB proteins are determined by western blotting. The results show that sodium propionate significantly inhibits the phosphorylation of NF-κB (Figure 7).

## Discussion

Mastitis is a major disease affecting dairy animal worldwide. It is characterized by mammary gland edema, mammary alveolar damage, and inflammatory cell infiltration. Propionate is a SCFA that is abundant as butyrate in the gut and blood, has been reported to have anti-inflammatory activities. In this study, we found that sodium propionate obviously ameliorate mammary tissues injury and MPO activity. These results indicate that sodium propionate has a protective effect on LPS-induced mastitis.

Mastitis is often associated with breakdown of the blood–milk barrier, leading to local systemic effects. Intramammary infusion with LPS has been used to mimic bacterial invasion and subsequent inflammatory responses in mammary glands (22, 23). The blood–milk barrier is an important physical barrier that provides important protection for milk integrity and the health of pups (24). However, during mastitis, the blood–milk barrier can become leaky. TJs contribute to restrict and modulate the permeability of the blood–milk barrier. In this study, we focus on occluding and claudin-3, which are the predominant factors that determine the permeability of TJs (25). It was found that sodium propionate upregulate the expressions of TJs. Furthermore, the leakage of FITC albumin from the interstitial side into the alveolar lumen is observed. The results show that pretreatment with sodium propionate can reduce the leakage of FITC albumin into the alveolar lumen. A more previous study also showed that SCFAs, especially butyrate and propionate, decrease TJ permeability in Caco-2 intestinal monolayer cells *via* lipoxygenase activation (26). Elamin et al. also reported that pretreatment with 4 mM/l propionate significantly alleviated the ethanol-induced barrier dysfunction, TJ and F-actin disruption, and metabolic stress in Caco-2 cell monolayers (27). Consistently, our results demonstrate that treatment of sodium propionate can restore the blood–milk barrier function through inhibiting the downregulation of TJ and the leakage of FITC albumin in LPS-induced mastitis in mice. It suggests that sodium propionate may have a potential application in attenuating the disruption of blood–milk barrier after mastitis, which needs to be further studied.

Mammary epithelial cells participate in the innate defense of the udder (28). LPS, a pathogen-associated molecular pattern, can elicit mastitis by *Escherichia coli* (29) and trigger an innate immune response with release of pro-inflammatory cytokines (30, 31). Thus, we used the mammary epithelial cells to investigate the mechanism of sodium propionate on LPS-induced mastitis *in vitro*. The role of cytokines in the pathophysiology of mastitis has been the subject of many studies. Cytokines are crucial factors involved in regulating immune response against different infections (32). Results obtained in this study reveal that sodium propionate significantly reduce the production of TNF-α, IL-1β, and IL-6 in a dose-dependent manner. Moreover, cytokines mediated restructuring of TJs proceeds through various signaling pathways to effect paracellular permeability (33). It suggests that decrease in pro-inflammatory cytokines may be attributed to the treatment of mastitis.

Numerous lines of evidence indicate that NF-κB play a pivotal role in the inflammatory responses through modulating the expressions of inflammatory genes (34). Our previous studies have demonstrated that NF-κB is involved in the pathogenesis of mastitis (19, 35). It has been proposed that through targeting NF-κB it may be possible to suppress inflammation development in the mammary glands. In this study, we examine a mechanism by which sodium propionate influences NF-κB activation in LPS-stimulated mMECs. Our results show that sodium propionate inhibit NF-κB phosphorylation.

Propionate is one of the main SCFAs, and it can act as an inhibitor of HDAC (36). Recently, it is reported that HDAC inhibitors modulated the activity of NF-κB in a number of different cell types including colonic epithelial cell lines and macrophages isolated from the lamia propria of the colon (18, 37, 38). We next investigate whether sodium propionate acts as a HDAC inhibitor to exert its anti-inflammatory effects. Similar to TSA, sodium propionate increased the overall levels of histone H3 acetylation. In primary mouse microglia, HDAC inhibitors affected the LPS-induced inflammatory responses by suppressing cytokines secretion (39). In this study, we observe that both TSA and sodium propionate induce the production of inflammatory cytokines (TNF-α, IL-1β, and IL-6), suggesting that sodium propionate may regulate inflammatory responses through HDAC inhibition.

In summary, this study demonstrate that sodium propionate exerts beneficial effects on improving the blood–milk barrier function, modulating inflammatory responses *via* the inhibition of HDAC, which, in turn, inhibits LPS-induced NF-κB activation and inflammatory cytokines production. Our study not only provides *in vivo* evidence but also gains underlying mechanistic insights into the potential therapeutic benefits of sodium propionate for the management of mastitis.

## Author Contributions

JW performed experiments and analyzed the data. YF and ZY conceived and designed the experiments. YF, ZY, and JW interpreted the data and wrote the paper. The authors sincerely thank from ZW, XZ, YW, YF, and ZY for their support during the study.

## Conflict of Interest Statement

The authors declare that the research was conducted in the absence of any commercial or financial relationships that could be construed as a potential conflict of interest.
